# Molecular Evolution of a Pervasive Natural Amino-Acid Substitution in *Drosophila cryptochrome*

**DOI:** 10.1371/journal.pone.0086483

**Published:** 2014-01-24

**Authors:** Mirko Pegoraro, Shumaila Noreen, Supriya Bhutani, Avgi Tsolou, Ralf Schmid, Charalambos P. Kyriacou, Eran Tauber

**Affiliations:** 1 Department of Genetics, University of Leicester, Leicester, United Kingdom; 2 Department of Molecular and Cellular Neurosciences, National Brain Research Centre, Manesar, Haryana, India; 3 Department of Molecular Biology and Genetics, Democritus University of Thrace, Alexandroupolis, Greece; 4 Department of Biochemistry, University of Leicester, Leicester, United Kingdom; North Carolina State University, United States of America

## Abstract

Genetic variations in circadian clock genes may serve as molecular adaptations, allowing populations to adapt to local environments. Here, we carried out a survey of genetic variation in *Drosophila cryptochrome* (*cry*), the fly’s dedicated circadian photoreceptor. An initial screen of 10 European *cry* alleles revealed substantial variation, including seven non-synonymous changes. The SNP frequency spectra and the excessive linkage disequilibrium in this locus suggested that this variation is maintained by natural selection. We focused on a non-conservative SNP involving a leucine - histidine replacement (L232H) and found that this polymorphism is common, with both alleles at intermediate frequencies across 27 populations surveyed in Europe, irrespective of latitude. Remarkably, we were able to reproduce this natural observation in the laboratory using replicate population cages where the minor allele frequency was initially set to 10%. Within 20 generations, the two allelic variants converged to approximately equal frequencies. Further experiments using congenic strains, showed that this SNP has a phenotypic impact, with variants showing significantly different eclosion profiles. At the long term, these phase differences in eclosion may contribute to genetic differentiation among individuals, and shape the evolution of wild populations.

## Introduction

The circadian clock consists of an evolutionarily conserved genetic network that drives the daily oscillations of a significant proportion of the transcriptome, in various organisms [1]–[3]. The covert rhythm of the clock is generated by an endogenous negative feedback mechanism [4], but this rhythm is entrained by external cues such as temperature and light, which exhibit regular daily cycles. Wild populations in different environments are therefore expected to show molecular adaptations of the clock to local conditions [5]. Furthermore, because day-length and temperature change gradually along latitude, any allelic variation in clock genes that follows a latitudinal cline is a strong candidate for an adaptive polymorphism that is driven by natural selection. The threonine-glycine repeat length polymorphism within the *period* gene of *Drosophila melanogaster* is a well-studied example of such a latitudinal cline in a clock gene [6]. This polymorphism is under balancing selection probably due to the different circadian temperature compensation properties that are determined by the various length alleles [5].

Another natural polymorphism in *Drosophila* that follows a cline in Europe was identified in the *timeless* (*tim)* gene [8], which encodes core clock protein that is important for circadian light resetting. This polymorphism involves a single-base insertion/deletion, situated between two alternative translation starts [7], resulting in two alleles of *tim* with substantial impact on circadian and seasonal photoresponsiveness that would be expected to be adaptive in temperate environments such as Europe [9]. However, this variation in *tim* appears to be driven by directional and not balancing selection, with the cline reflecting the recent spread of the new *tim* allele from southern Europe where it may have originated [8]. The impact of the new *tim* allele on seasonal behaviour underscores the notion that core clock genes, particularly those that channel sensory information to the pacemaker, provide an optimal target for natural selection.

CRYPTOCHROME (CRY) is an evolutionary conserved blue-light photoreceptor which is associated with the circadian system in a broad range of organisms including mammals [10], insects [11], [12] and plants [13]. In mammals, CRY serves as a core clock protein that heterodimerizes with PER proteins [14]–[16]. In *Drosophila,* CRY is the dedicated circadian photoreceptor, as demonstrated by *cry* null mutants being rhythmic in constant light, a condition that normally causes wild-type flies to become arrhythmic [17], [18]. However, in peripheral clocks fly CRY appears to assume a more central function, more similar to its role in the mammalian clock [19]. Here, we carried a survey of genetic variation in *cry* alleles from European and North-American *D. melanogaster* populations, and attempted to tease out the evolutionary dynamics of this genomic regions as well as testing for any possible functional role among the major *cry* variants.

## Materials and Methods

### Fly Strains

*D. melanogaster* isofemale lines were established from natural populations collected across Europe (Table S1). Flies homozygous for each natural *cry* allele *(cry-H* and *cry-L)* were established from a single isofemale line from a natural population in Rende Italy. Consequently the genetic background would be expected to be randomised around the *cry* locus in these flies. Sequencing of these strains revealed additional non-synonymous SNPs at positions C3957T (P218L), C4310G (D335E) and A4347G (N348D), using the FlyBase reference sequence FBgn0025680. We also generated another natural congenic background by repeatedly backcrossing an inbred strain from Naturno, Italy carrying *cry-L* to Canton-S strain *(cry-H)* for 10 generations using PCR to genotype recombinants (see below).

Transgenic flies were generated by modifying the construct that was used to generate the *UAS-HAcry* strain [20], which encodes the *cry-L* variant. A 914 bp fragment digested by PflFI-BstEII was replaced with a fragment carrying the *cry-H* variant, amplified by PCR from a natural isofemale line. The transgenic constructs differ only in the L232H variation. Transgenic strains were generated by *P*-element transformation, and four *cry-H* independent insertions were isolated and compared against three *UAS-cry-L* strains. Another strain, carrying both *cry-L* and *cry-H* construct (homozygous) was generated to simulate a heterozygous genotype. The *UAS* strains were driven using *cry-Gal4-16* (Bloomington stock 24514).

### L232H Genotyping

DNA was isolated from single fly according to Gloor et al. [21]. For genotyping, we used mismatch PCR using the forward L and H primers (*5′- AGAAACACAGGCCTTAGTT-3′*; *5′-AGAAACACAGGCCTTAGAT-3′* position 1982) to specifically amplify a single band of 1195 bp when coupled with a common reverse primer (*5′-GTACTCCTTCAAACCACCA-3′* position 3177). PCR products were visualized on 1.5% agarose gels.

### Western Blot

Three day old *cry-H* [CS] or *cry-L* [CS] flies were placed in plastic vials (10x2 cm) containing sugar food (4.6% sugar, 4.6% brewer’s yeast, 1.25% agar, 0.2% methyl 4-hydroxybenzoate). Vials were placed in incubator for four days at 25°C in LD 12∶12. During the fourth day, flies were collected every 3 h beginning at ZT1 (one hour after lights on) and immediately frozen in liquid nitrogen. Proteins were isolated from 40–50 heads from each time point as in Edery *et al*. 1994 [22]. The protein preparation were separated in 8% polyacrylamide gels (37.5∶1, acrylamide:bisacrylamide ratio; Sigma Aldrich-USA). Blots were incubated with a CRY antiserum raised against N terminal residues 1–188 (1∶1000, [48]) and mouse anti-tubulin (1∶5000, abcam-UK) diluted in TBST-5% dry milk. An anti- guinea pig IgG-HRP (1∶8000; Amersham-UK) and an anti-mouse IgG-HRP (1∶2000; Sigma Aldrich-USA) were used as secondary antibodies. Visualization of CRY and TUB was performed by chemiluminescence (ECL Advance Western Blotting Detection Kit, GE HEALTHCARE) and autoradiography. Three blots for each line were analysed using Image J software (version 1.45b) for the quantification of the signals. Background signal was subtracted, normalised by the TUB signal, and expressed as % of maximum signal in each plot.

## Results

### Molecular Variation

**Figure 1:**
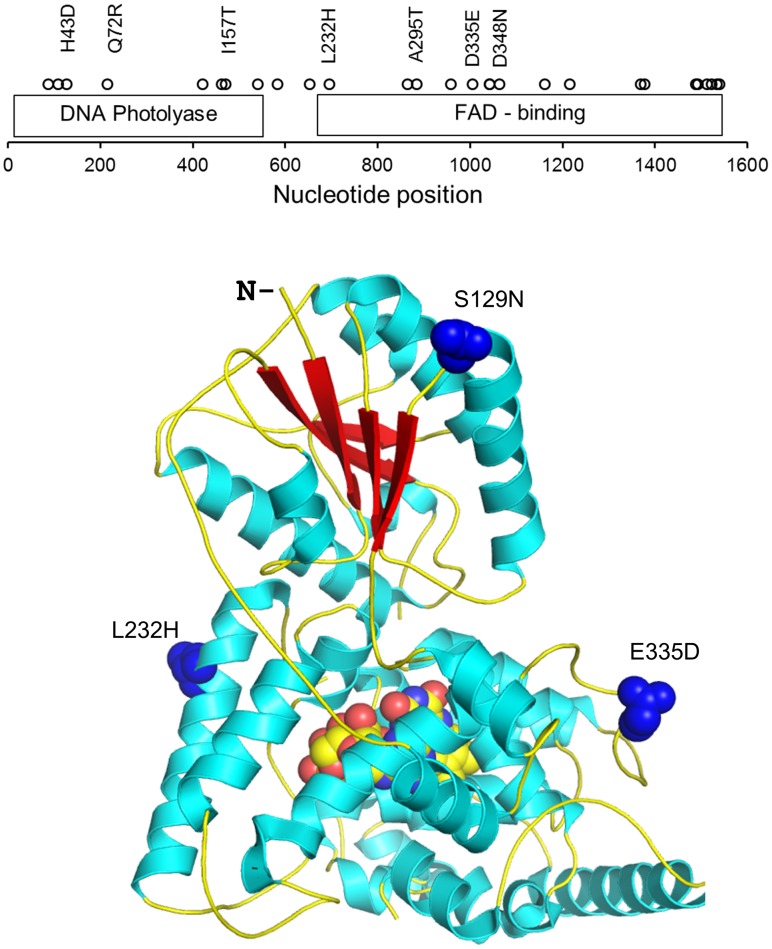
Natural genetic variation in *Drosophila cry.* A. Polymorphisms found in the coding DNA (CD) of *cry* are indicated (circles) in 10 European alleles. Replacement SNPs are also depicted. Below, the L232H SNP is mapped onto the crystal structure of dCRY together with two other SNPs which are in strong linkage. All SNPs are located on the protein surface, away from the FAD pocket.

Our initial survey of the complete protein-coding sequence (CD) of 10 natural *cry* alleles from various European wild populations (Table S1) revealed an extensive amount of genetic variation, including 21 silent single-nucleotide polymorphisms (SNPs) and 7 replacement SNPs, four of which mapped to the FAD binding domain of the protein (Fig. 1; Fig. S1). One of these SNPs encoded a radical leucine-histidine change (L232H), with both alleles showing intermediate frequencies. Analysis of a North American population sample from Raleigh in North Carolina (n = 35), sequenced by the *Drosophila* Population Genomics Project (DPGP), revealed similar extensive variation in *cry*, with 29 silent and 17 replacement changes in the coding DNA. Nucleotide diversity in the coding DNA, estimated using (i) θ_s,_ the number of segregating sites [23] was 0.00765 (0.00249 s.d.), and based on (ii) π, the average number of nucleotide differences between pairs of sites [24] which was 0.00649 (0.00051 s.d.). The L232H polymorphism was also present in the Raleigh population sample (frequency of *cry-H* variant, the minor allele, is 18/35), and interestingly, was also segregating in the small population sample from Malawi (*cry-H* variant 2/5), suggesting an ancient polymorphism.

We took advantage of the recently published crystal structure of CRY [25] to visualise the putative effect of the SNP variants on the protein (Fig. 1B). L232H (as well as two linked SNPs) mapped to the protein surface, away from the FAD-binding domain of the protein, and therefore unlikely to affect the redox status of the co-factor, which is thought to be important for the photoactivation of CRY [26], [27]. However, when we used the Site Directed Mutator (SDM) function [28] to test the effect of the L232H polymorphism on the protein stability (the algorithm uses environment-specific substitution tables to calculate free-energy difference between folded and unfolded structure), we found that the H variant is destabilised compared with the L variant (stability score ΔΔ*G* = −1.11). The other linked SNPs, S129N is also destabilised in the Ser allele (ΔΔ*G* = −2.32), but the SNP E135D showed no effect on protein stability (ΔΔ*G* : 0.01).

**Figure 2:**
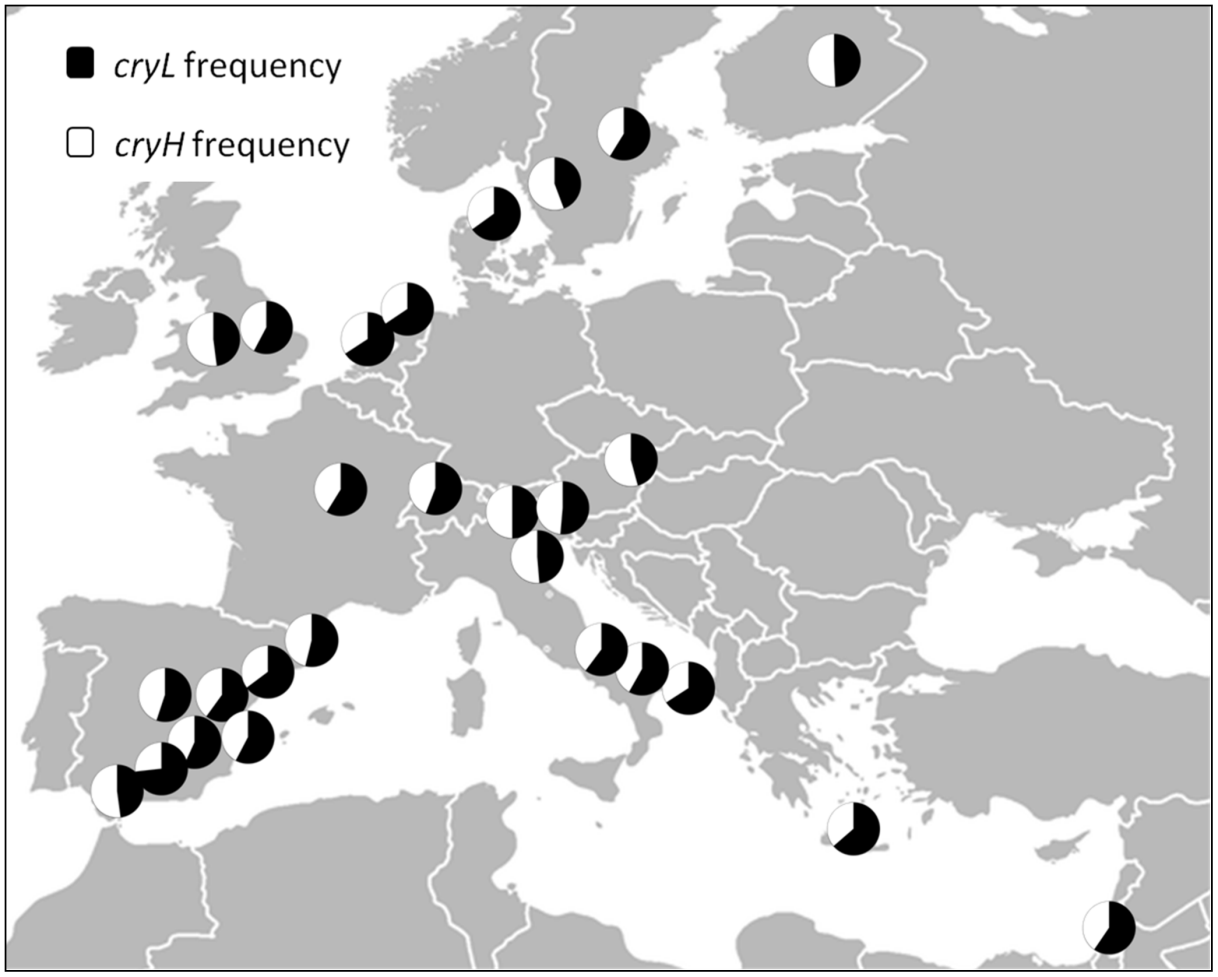
Spatial distribution of the L232H cry polymorphism.

To test the spatial distribution of the L232H polymorphism, we have genotyped this locus in 27 population samples across Europe (Fig. 2). While both alleles were present in all populations tested, their frequencies did not follow a latitudinal cline (F_1,25_ = 1.84, p = 0.19). The fact that both alleles have remarkably similar frequencies in all populations (95% CI of *cry-L* : 54%−60% ), despite the apparent ancient origin of this variation, suggested that this polymorphism may be under balancing selection.

We used the *cry* genomic DNA sequences from the 35 Raleigh alleles to test whether the variation observed is consistent with neutrality. Tajima’s D statistics [29] calculated for the whole region did not deviate from neutral expectation (Fig. S1), but a sliding window analysis [49] revealed both negative and positive significant peaks, the latter consistent with excess of intermediate frequency alleles under balancing selection. However, neither of these peaks co-localised with the focal polymorphism.

**Figure 3:**
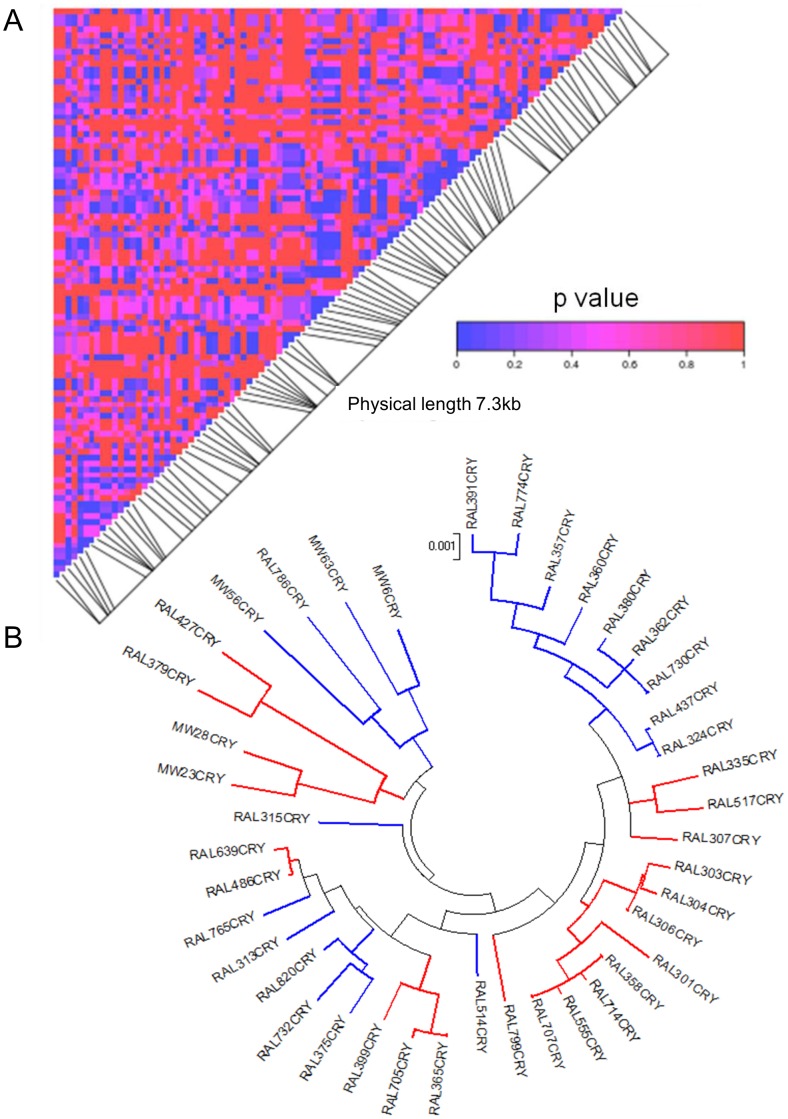
Linkage disequilibrium (LD) and haplotype structure of *cry* alleles. **A**. The significance of LD 7.3*cry* is depicted as a matrix of pairwise comparisons among 99 informative SNPs. P values (uncorrected for multiple testing) are shaded by level of significance (see colour code). **B**. Maximum Likelihood gene tree for *cry,* depicting L (blue) and H (red) haplotypes. The tree is drawn to scale, with branch lengths measured in the number of substitutions per site.

We observed excessive linkage-disequilibrium (LD) between polymorphic sites throughout the 7.3 Kb region encompassing *cry* (Fig. 3A). This is often a characteristic of balancing selection, where each allele accumulates a set of mutations at linked sites, resulting in excess LD among SNPs flanking the selected locus [50]. The testing of all pairwise combinations of 99 informative SNPs revealed 561 pairs with a significant LD (p<0.05 Fisher-exact test, 23 pairs remain significant following a Bonferroni procedure). The fact that LD was not restricted to adjacent sites, but also included remote sites (Fig. 3, 285 sites more than 1 Kb apart), even though LD usually decays quickly in *Drosophila* [30], argues against a neutral mutation-drift equilibrium in this region, and is one of the hallmarks of balancing selection [31]. However, the LD was scattered without apparent large blocks of high LD that would have been expected under long standing balancing selection. Similarly, the gene tree of *cry* (Fig. 3B) does not reveal two major clades (connected by long branches) that would be expected under balancing selection [32], presumably due to recombination and gene conversion that have eroded the haplotype structure.

In five *cry* alleles from the closely related species, *D. simulans*, only the *cry-L* variant was present, suggesting that *cry-H* is the derived variant. We compared the variation in *D. simulans* sequences (n = 5) with the *D. melanogaster* Raleigh alleles (n = 35) in a McDonald-Kreitman test [33]. We found 33 fixed and 56 polymorphic, silent changes (Ds,Ps), and 2 fixed and 23 polymorphic (Dn,Pn) replacement changes, resulting in a significant departure from neutrality (p = 0.006, Fisher-exact test). The ratio (Pn/Ps)/(Dn/Ds) known as the neutrality index (NI), was 6.7, reflecting the excess of polymorphic replacement (or deficiency of fixed replacements) in the data, which is consistent with either selection against slightly deleterious mutations, or strong balancing selection promoting the excess of polymorphic replacement changes [34]. We note however that previous examples for balancing selection such in *Adh* were associated with NI <1 [33], while cases where NI >1 (as in *cry*), for example in mtDNA genes are thought to be due to polymorphisms that are slightly deleterious [35].

We have also tested the extent of DNA divergence between the H and L sequences, using various measures of genetic differentiation [36]. We found evidence for significant population differentiation indicated by Kst (0.074, p<0.0001), Kst* (0.027, p<0.001), Z (259.9, p<0.0001), Z* (5.17, p<0.0001) and Snn [37] (0.76, p<0.01). These tests suggested that gene flow between the two haplotype groups is rather small, and that the two haplotypes could be considered two subpopulations.

### Functional Analyses

**Figure 4:**
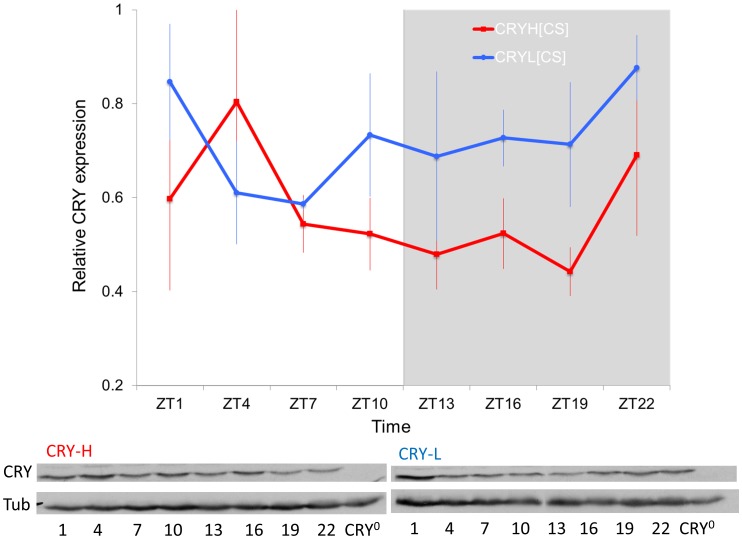
Expression of CRY variants. Top panel shows mean ± SEM TIM/TUB ratios from Western blots of CRYH (red) and CRYL (blue) in the CS genetic background lines (25°C in LD12∶12, n = 3 blots for each variant); relative expression refers to the percentage of maximum ratio obtained in each blot. Representative examples are shown at the bottom panel.

The expression of CRY under light-dark cycles was examined by Western analysis. CRY abundance differed significantly between natural congenic lines (CS background) carrying the *cry-H* or *cry-L* variants (Fig. 4, F_1,32_ = 5.97, P = 0.02). This was particularly evident during the dark phase, where expression of CRY-L was higher than CRY-H, a result consistent with the predicted increased stability of this variant (see SDM score above).

The free-running circadian period (in continuous darkness, DD) of locomotor activity was analysed in both natural variants (Rende background) and in transgenic male flies expressing hemizygous *UAS-cry-L* or *UAS-cry-H,* driven by *cry-GAL4*. There was a small, but significant difference in their circadian period (Fig. S2), with *UAS-cry-L* showing longer periods (F_2,142_ = 10.69, p<0.0001). Note that both lines showed unusually long periods (of 25–26.5 hr) that was attributable to the Gal4 transgene. The period of flies expressing both *UAS-cry-L* and *UAS-cry-H* transgenes (simulating a heterozygous genotype) were intermediate to the homozygotes. Control flies carrying only the *UAS* transgene (H or L) did not show any difference in their period (F_1,91_ = 0.35, p = 0.55). The circadian period in natural congenic flies (Rende background) showed a similar trend with a significant difference among genotypes (F_2,96_ = 5.78, p = 0.0042) and *cry-L* again having a significantly longer period than *cry-H*, but here, heterozygous flies resembled *cry-H* (Fig. S2).

The natural *cry* variants also showed differences in their activity profiles under light-dark cycle (LD), manifested as phase differences in the morning and the evening peaks of locomotor activity (Fig. S3). However, these differences were highly background dependent and differed between males and females. For example, in Rende background flies, *cry-L* homozygous males show the earliest morning peak, while in CS flies, males with this genotype are the latest (Fig. S3). In transgenic flies, control *UAS* strains exhibited phase differences (presumably because position effect of the insertion) and were therefore uninformative for this analysis (not shown).

**Figure 5:**
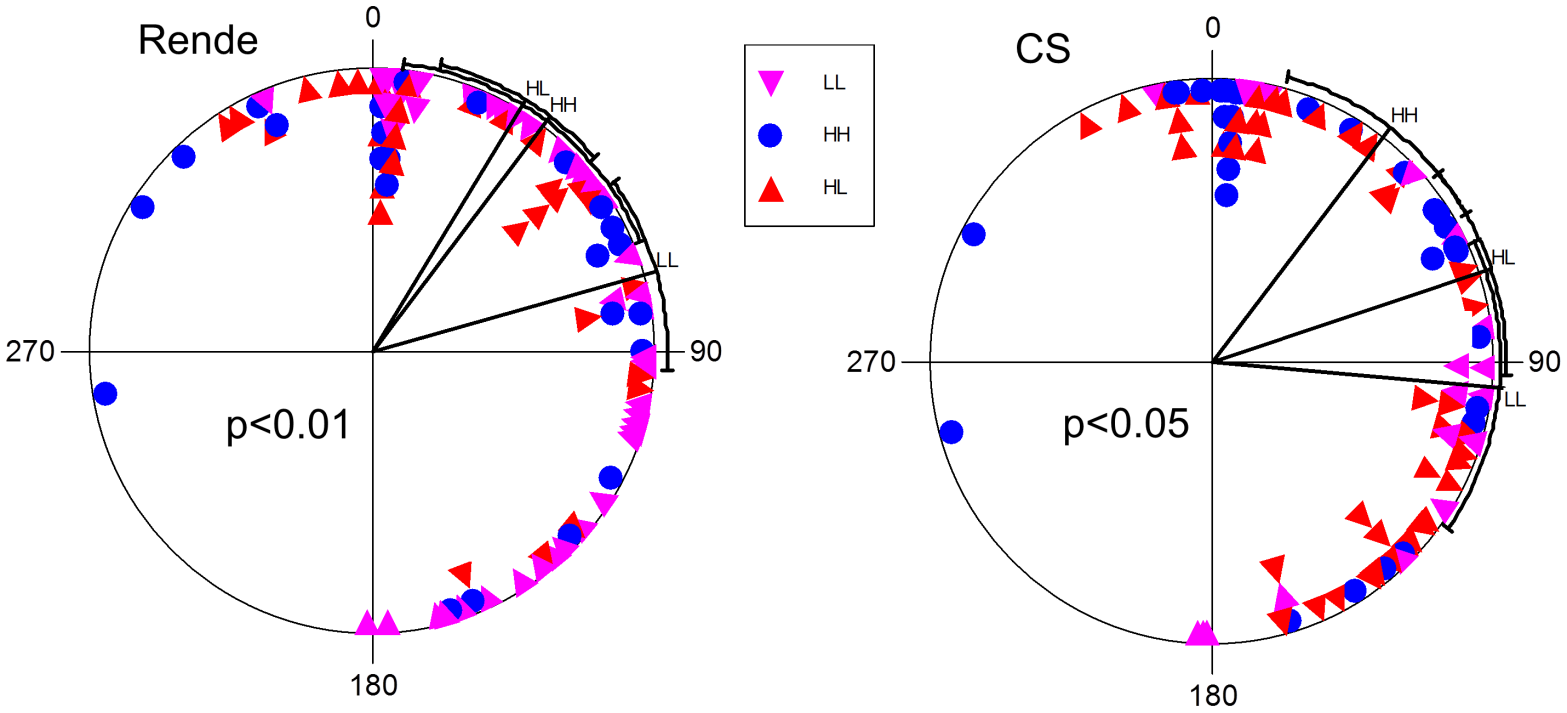
Phase variation in eclosion associated with the L232H polymorphism. In LD cycles, natural congenic strain females (Rende and CS background) show significantly different eclosion phase. The lines are the mean vectors and their direction indicates the mean phase (±95% confidence intervals). The sector 0–180° represents the light phase (Zt 0–12), while dark is between 181–360° (Zt 12–24). Each symbol represents two flies. The p-value for the difference between *HH* and *LL* genotypes using Watson-Williams F-test is also shown.

Given the possible role of CRY in the circadian regulation of eclosion [38] (but see [18], [39]), we sought to determine whether the L232H polymorphism is also important for this phenotype. In females, eclosion differed significantly between *cry-H* and *cry-L* variants (F_1,66_ = 4.56, p = 0.036), with *cry-H* females eclosing 150 min earlier (Fig. 5). The heterozygous females were significantly different from the *cry-L* variants (F_1,72_ = 9.78, p = 0.003), with eclosion phase similar to *cry-H* homozygous flies (Fig. 4). Similar results were exhibited by the CS congenic natural strains (F2,91 = 4.9 p<0.05). In males however, the difference in eclosion phase was not significantly different (not shown).

Although CRY has a major role in circadian photoresponsiveness [40] we did not find evidence for differences in light sensitivity between the CRY variants neither biochemically nor behaviourally (Fig. S4). For example, similar binding of each of the CRY variants to TIM was measured by the yeast two hybrid system, and flies carrying the different variants showed similar behavioural phase shifts in light pulse experiments (Fig. S4).

### Experimental Evolution

**Figure 6:**
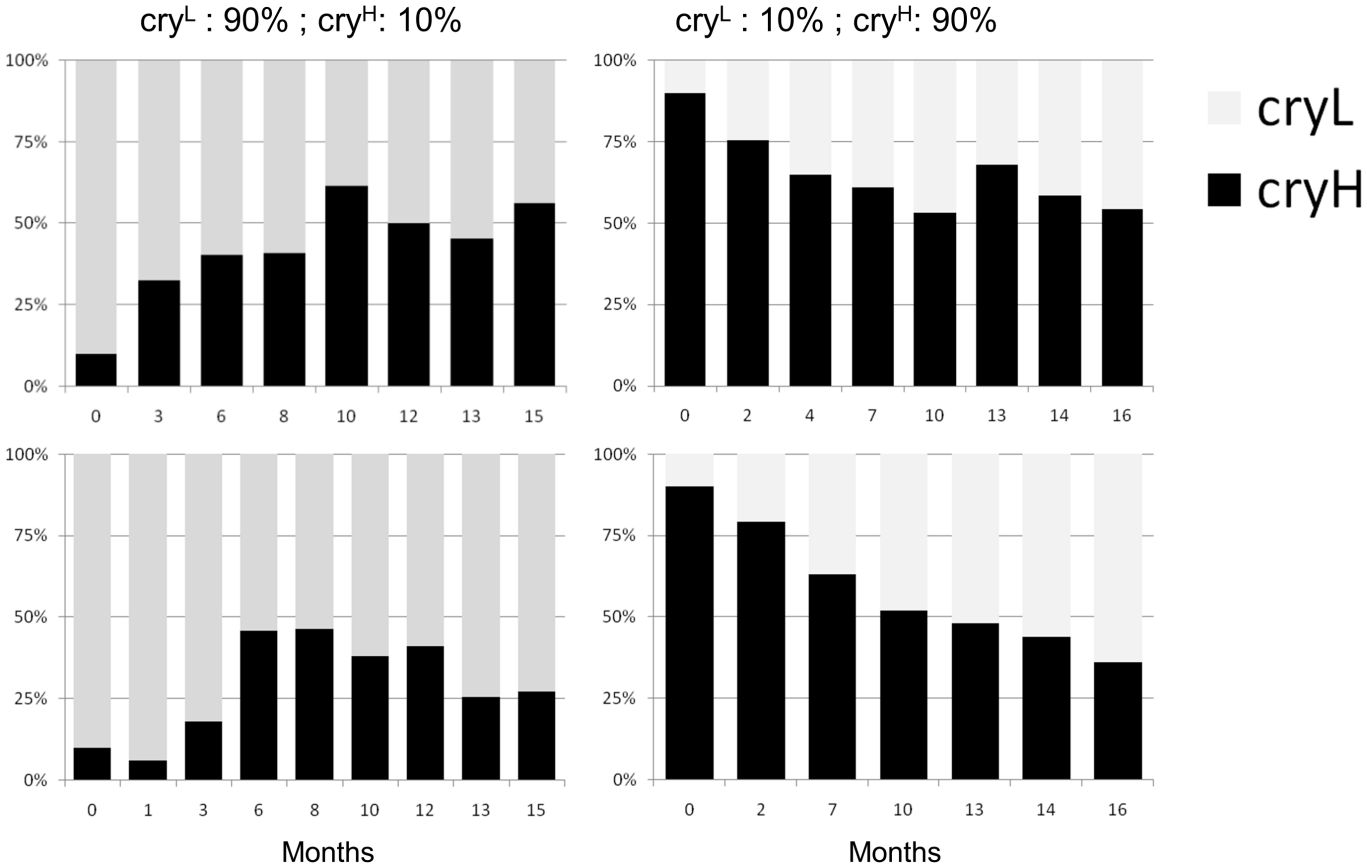
Experimental evolution of L232H polymorphism. Changes in allele frequencies in four population cages (Ne = 1000–2000) are depicted. 500 individuals of known genotype were used to initiate each population and the initial allele frequencies are shown above. Subsequently allele frequencies were monitored by PCR genotyping.

To demonstrate the non-neutral dynamics of the L232H SNP we initiated four experimental populations using our natural congenic strains (Rende background, n = 500). The founding populations were designed to have largely biased allele frequencies; two population replicates with *cry-L*: 90% *cry-H* :10%, and two replicates with the reciprocal frequencies (note that the natural congenic lines carried two additional replacement SNPs which are in strong LD with L232H, see Methods). When we genotyped samples of ∼50 flies extracted from the population of 1–2000 individuals, we observed a remarkable convergence to intermediate allele frequencies in all cages (Fig. 6): within 10 months (∼20 generations), the minor allele frequencies in all cages climbed from 10% to 38–50% (p<0.0001, Fisher exact test, for all cages). These intermediate frequencies were further maintained so that by the end of the experiment (16 months) minor allele frequencies were higher than the initial frequencies, suggesting that this polymorphism is driven by a non-neutral process. Intriguingly, analysis of genotype frequencies (Fig. S5) indicated a significant departure from Hardy-Weinberg equilibrium only in two cages (in both cases, this has occurred at the early stages of the experiment).

## Discussion

Natural genetic variation in *Drosophila* circadian clock genes has been previously studied in *per* and *tim* [41]. The polymorphism in *per* follows a latitudinal cline in Europe [6], driven by balancing selection, [42], and probably related to the different variants’ responses to temperature changes [5]. The *tim* polymorphism has a latitudinal component but is really a distance cline with the novel variant spreading by directional selection from its point of origin in south-eastern Italy [8]. The *tim* variants’ differential light responses in both their circadian behaviour and photoperiodic diapause mechanisms suggest a possible seasonal adaptation that may drive the positive selection of the new variant in the temperate European environment [8], [9]. In contrast to *per* and *tim* polymorphisms, the *cry* variants we have identified do not show a spatial dimension to their frequency distribution in Europe and are maintained at intermediate frequencies over a wide range of latitudes. The L232H polymorphism we have identified represents an ancient polymorphism, which is also segregating in African populations.

The fact that this polymorphism appears to be ubiquitous suggests that this variation is not neutral and is consistent with the signature for balancing selection we find in this genomic region (e.g. strong LD, significant MK test and genetic differentiation between haplotype groups). Furthermore, the uniform spatial distribution of the *L232H* alleles may indicate that any functional role of this SNP is neither light nor temperature related otherwise a latitudinal cline might be expected. Indeed, we did not find any consistent evidence for differential circadian behaviour or photosensitivity in flies carrying the different *cry* variants. We did observe differences in levels of CRY expression in the two variants, which had been predicted based on structural considerations to affect the stability of CRY. However, these stability differences do not appear to affect circadian photoresponsiveness neither in behavioural light-pulse experiments nor in yeast assays testing the dimerization of CRY with TIM under light. It is however still possible those other SNPs in *cry* contribute to variation in photoresponsiveness, which were not captured in our screen. Indeed the pattern of variation, as reflected by the Tajima and MK test, might be consistent with multiple SNPs that are targets for selection in this gene, and associated with different phenotypes.

We did however identify significantly different eclosion phases in flies carrying the different *cry* variants under light entrainment, (Fig. 5). This resembles the situation for the *per* gene, where we have previously shown that interspecific variation in this gene alone can drive different temporal locomotor and mating profiles, potentially contributing to temporal speciation [43]. The phase variation in the *cry* variants might similarly serve as a mechanism to drive temporal partitioning within an ecological niche. Indeed, variation in *cry* alleles was suggested to cause differences in mating time between populations of the melon fly, *Bactrocera cucurbitae,* leading to premating reproductive isolation [44]. Thus, diurnal phase variation encoded by different clock alleles may serve as the first step leading to temporal speciation, followed by accumulation of pre- and postzygotic barriers between flies carrying the different alleles. The power of this mechanism is that no assumption regarding the fitness of each of the allele is required, merely a phase difference. The fact that the L and H haplotype showed a significant evidence for genetic differentiation as indicated by nucleotide sequence-based statistics (e.g. Ks, Snn), suggests that the two groups of haplotypes may be considered as sub-populations (i.e. limited gene flow), which is consistent with such a mechanism. A recent study also showed extremely high genetic differentiation in *cry* among *D. melanogaster* populations in Australia [45], which would also support this circadian speciation model.

The experiments with the population cages suggested that the mechanism that maintains both alleles at similar frequencies, can be recapitulated in the laboratory (Fig. 6). Thus, although heterogeneous selection may contribute to maintaining the *L232H* polymorphism in the wild, our experimental evolution suggests that fitness benefits of each allele at a different environment is an unlikely mechanism for underlying this polymorphism. Assortative mating (driven by phase differences, as described above), or increased fitness of heterozygous individuals, represent other mechanisms that might provide the basis for maintenance of this polymorphism. However, these two mechanisms would have been reflected in either reduced or increased frequency of the heterozygotes, but the genotype frequencies we have sampled did not depart significantly from Hardy-Weinberg Equilibrium. The *L232H* polymorphism may also be maintained by negative frequency-dependent selection, where the frequency of a given allele is decreased as its frequency increases [46]. This mechanism has been identified as the basis for the natural polymorphism in the *foraging* gene in *Drosophila* [47]. To what extent a frequency-dependent mechanism contributes to the polymorphism in *cry* is yet to be determined. It is also important to note that our natural congenic lines carried additional replacement SNPs (see Methods), two of which are in strong LD with L232H that might contribute to the phenotypic variation. It is also possible that variations in flanking regions to *cry* could explain the maintenance of the *cry* polymorphism, although this is less likely due to the introgression process that was used to generate these strains. Overall, we have identified a few phenotypic phase differences, but their link to the L232H polymorphism, and how this variation is maintained, remain obscure. A detailed analysis of the phenotypes and fitness of each of the *cry* variants accompanied by modelling the allele dynamics, will allow a fuller understanding of the evolution of this polymorphism as well as its role in temporally regulated phenotypes.

## Supporting Information

Figure S1 **The Tajima’s D statistic across the genomic region of** ***cry*** **for the Raleigh population.** The figure shows a sliding window of Tajima’s D calculated every 200 bp, with window size of 25 bp. The 95% confidence intervals were produced by coalescent simulation using θ = 36.17, n = 35, number of replications: 1000, under either no recombination (red line), or intermediate recombination, R per gene of 10.00 (blue line). The coding DNA is shown at the bottom (grey bars), and the L232H SNP is indicated by an orange circle.
(TIF)

Figure S2 **Circadian period of** ***cry*** **variants.** Boxplot showing median (black line within box), first and third quartiles (box boundaries) of the circadian period measured in DD. Transgenic flies (upper panel) expressing *cryGAL4/cry^b^* (Gcb), *cryGAL4/UAS-cryH (GH), cryGAL4/UAS-cryL (GL),* or both *UAS* transgenes (GHL). The UAS controls *UAS-cryH/cry^b^* (Hcb), *UAS-cryL/cry^b^* (Lcb), or both (HLcb) are also shown. Congenic strains (Rende background) are depicted below. Different letters indicate significant differences at the p<0.05 level, according to Tukey post-hoc test.
(TIF)

Figure S3 **Phase variation in locomotor activity.** In LD cycles, natural congenic strains show significant different onset of morning (left) and evening activity bouts in males (top panel) and females (bottom panel). The lines are the mean vectors and their direction indicates the mean phase (±95% confidence intervals). Each symbol represents two flies. The sector 0–180° represents the light phase (Zt 0–12), while dark is between 181–360° (Zt 12–24). The p-value for the difference between *HH* and *LL* genotypes using the Watson-Williams F-test is also shown. Data are shown for Rende and Canton-S backgrounds.
(TIF)

Figure S4 **Circadian photosensitivity of** ***cry*** **variants.** Behavioural phase delay to a 20 min light pulse administered at ZT15 of RENDE (**A**), CS (**B**) or transgenic (**C**) lines expressing CRYH, CRYL or both. Delays in hours (assigned negative values by convention) were converted to degrees (1 hr = 15°). No statistical difference was found in the delay response of flies expressing CRYH or CRYL (Watson-Williams F-test, RENDE F_2,80_ = 1.148 p = 0.322, CS F_2,61_ = 0.509 p = 0.604, Transgenic F_2,209_ = 1.111 p = 0.331). The lines represent the mean vectors (±95% confidence intervals). **D**. Yeast two hybrid testing of TIM-CRY interactions. OD420 normalized over OD720 of 20 yeast colonies expressing either L-TIM or S-TIM with CRYH or CRYL. No difference was found between the interaction of CRYH and CRYL with either L-TIM or S-TIM (L-TIM CRYH vs CRYL F_1,38_ = 1.038 p = 0.3146; S-TIM CRYH vs CRYL F_1,38_ = 0.06044 p = 0.8071).
(TIF)

Figure S5 **Change in genotype frequencies of the L232H SNP in population cages.** Ternary plots showing the genotype frequency in each of the four population cages (same arrangement as in Fig. 5). Populations were genotyped 6 times during the experiment (16 months). Genotype frequencies under Hardy-Weinburg Equilibrium (HWE) are within the 95% limits and shown in green. Significant departures from HWE are shown in red.
(TIF)

Table S1 **Fly population samples.** Geographical information, number of iso-female lines (N), and allele frequency.
(DOC)
