# Supplementary figures and images for: Molecular Evolution of a Pervasive Natural Amino-Acid Substitution in Drosophila cryptochrome
Source: PLoS One. 2014 Jan 24;9(1):e86483. doi: 10.1371/journal.pone.0086483 (PMC3901690; doi:10.1371/journal.pone.0086483)

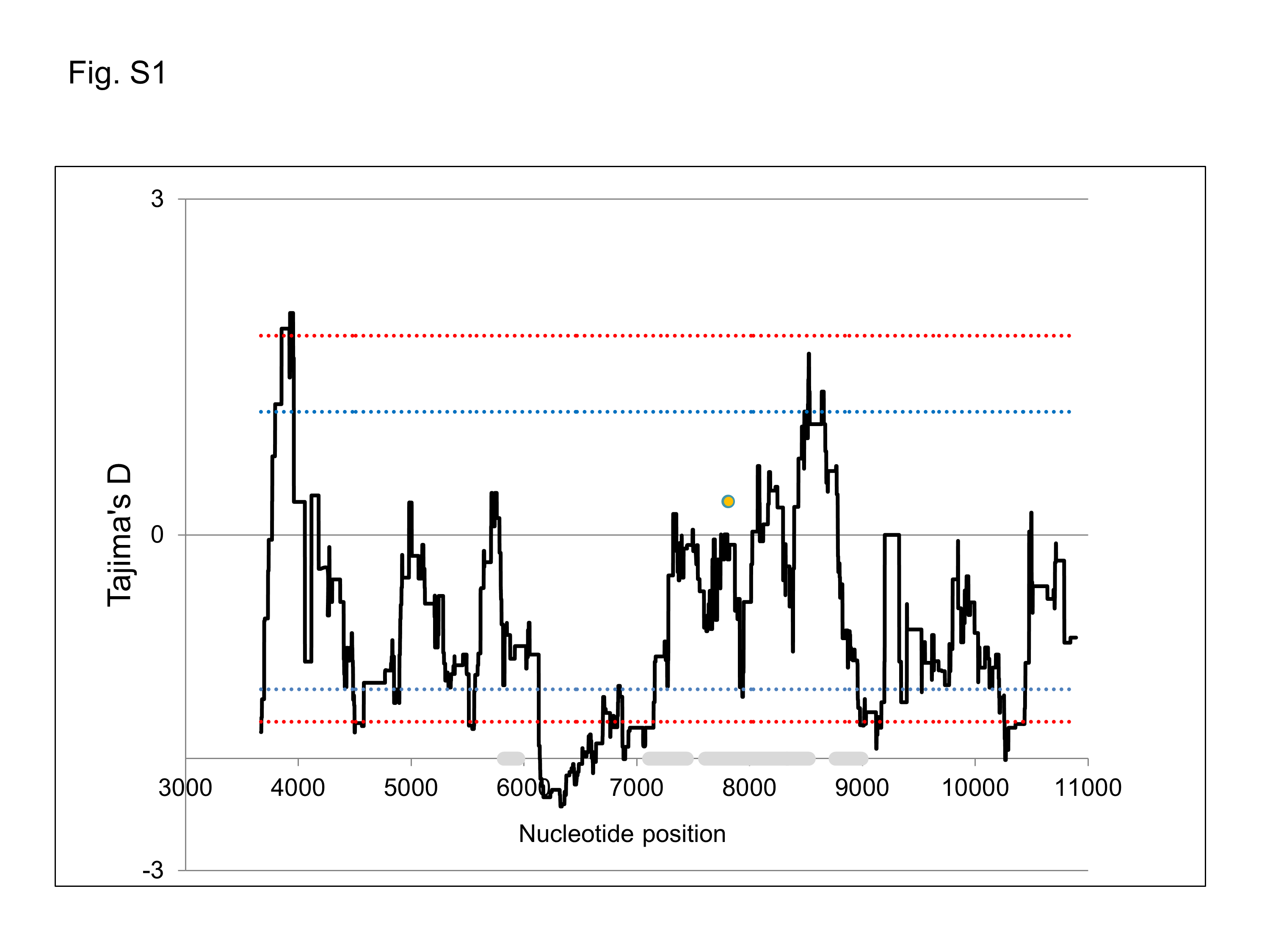

Supplement: Figure S1 — The Tajima’s D statistic across the genomic region of cry for the Raleigh population. The figure shows a sliding window of Tajima’s D calculated every 200 bp, with window size of 25 bp. The 95% confidence intervals were produced by coalescent simulation using θ = 36.17, n = 35, number of replications: 1000, under either no recombination (red line), or intermediate recombination, R per gene of 10.00 (blue line). The coding DNA is shown at the bottom (grey bars), and the L232H SNP is indicated by an orange circle. (TIF) [file pone.0086483.s001.tif]

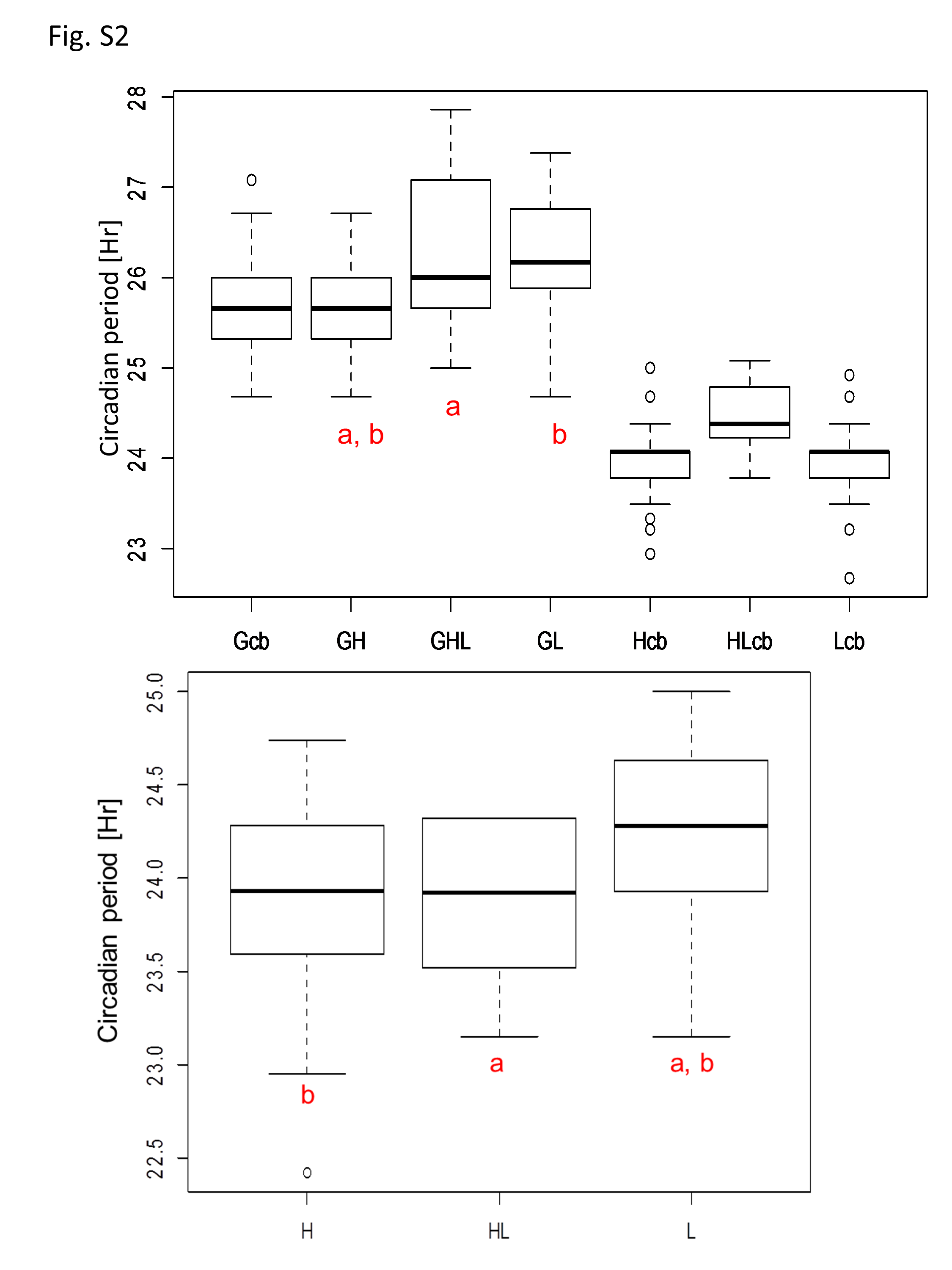

Supplement: Figure S2 — Circadian period of cry variants. Boxplot showing median (black line within box), first and third quartiles (box boundaries) of the circadian period measured in DD. Transgenic flies (upper panel) expressing cryGAL4/cryb (Gcb), cryGAL4/UAS-cryH (GH), cryGAL4/UAS-cryL (GL), or both UAS transgenes (GHL). The UAS controls UAS-cryH/cryb (Hcb), UAS-cryL/cryb (Lcb), or both (HLcb) are also shown. Congenic strains (Rende background) are depicted below. Different letters indicate significant differences at the p<0.05 level, according to Tukey post-hoc test. (TIF) [file pone.0086483.s002.tif]

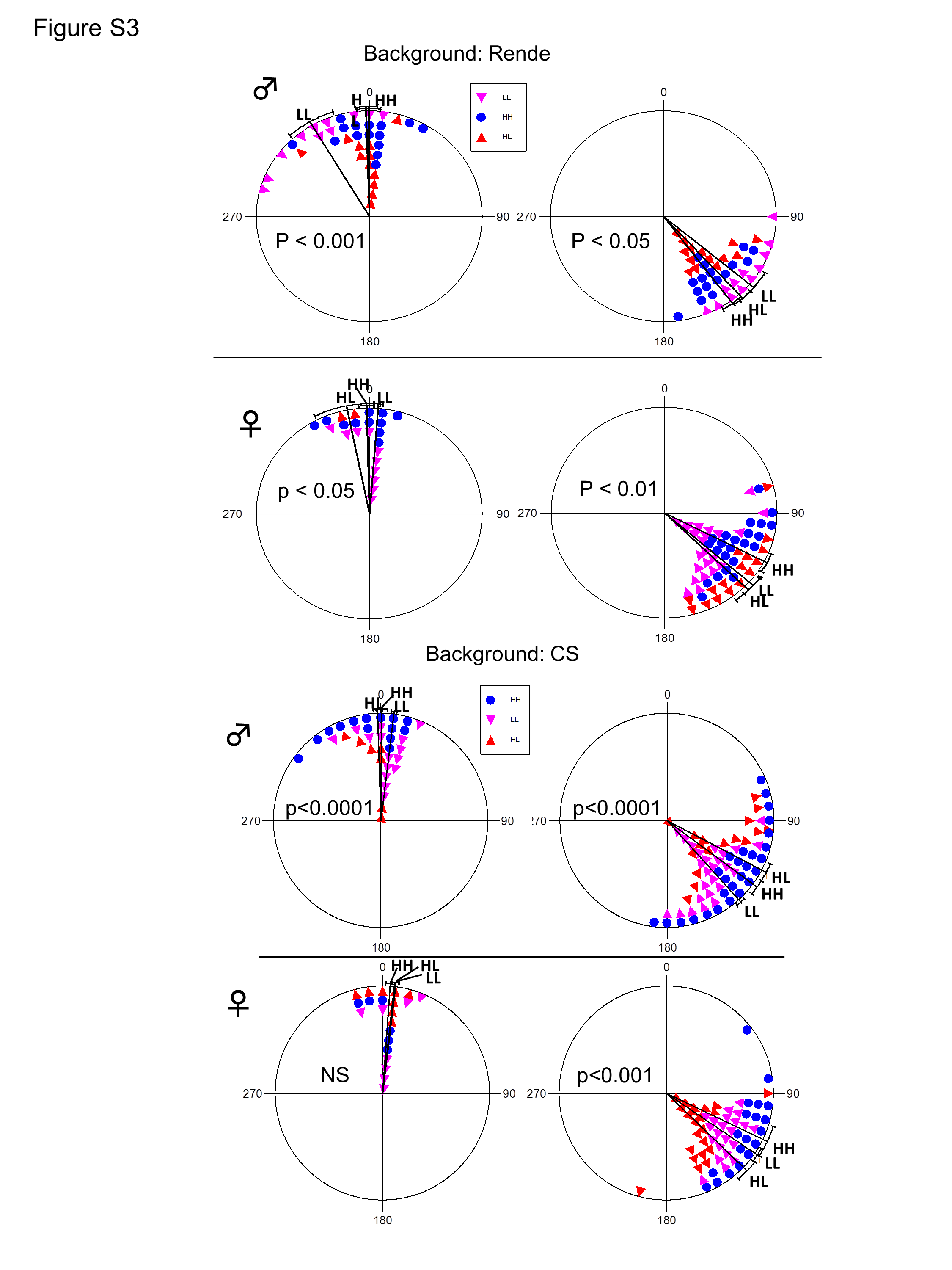

Supplement: Figure S3 — Phase variation in locomotor activity. In LD cycles, natural congenic strains show significant different onset of morning (left) and evening activity bouts in males (top panel) and females (bottom panel). The lines are the mean vectors and their direction indicates the mean phase (±95% confidence intervals). Each symbol represents two flies. The sector 0–180° represents the light phase (Zt 0–12), while dark is between 181–360° (Zt 12–24). The p-value for the difference between HH and LL genotypes using the Watson-Williams F-test is also shown. Data are shown for Rende and Canton-S backgrounds. (TIF) [file pone.0086483.s003.tif]

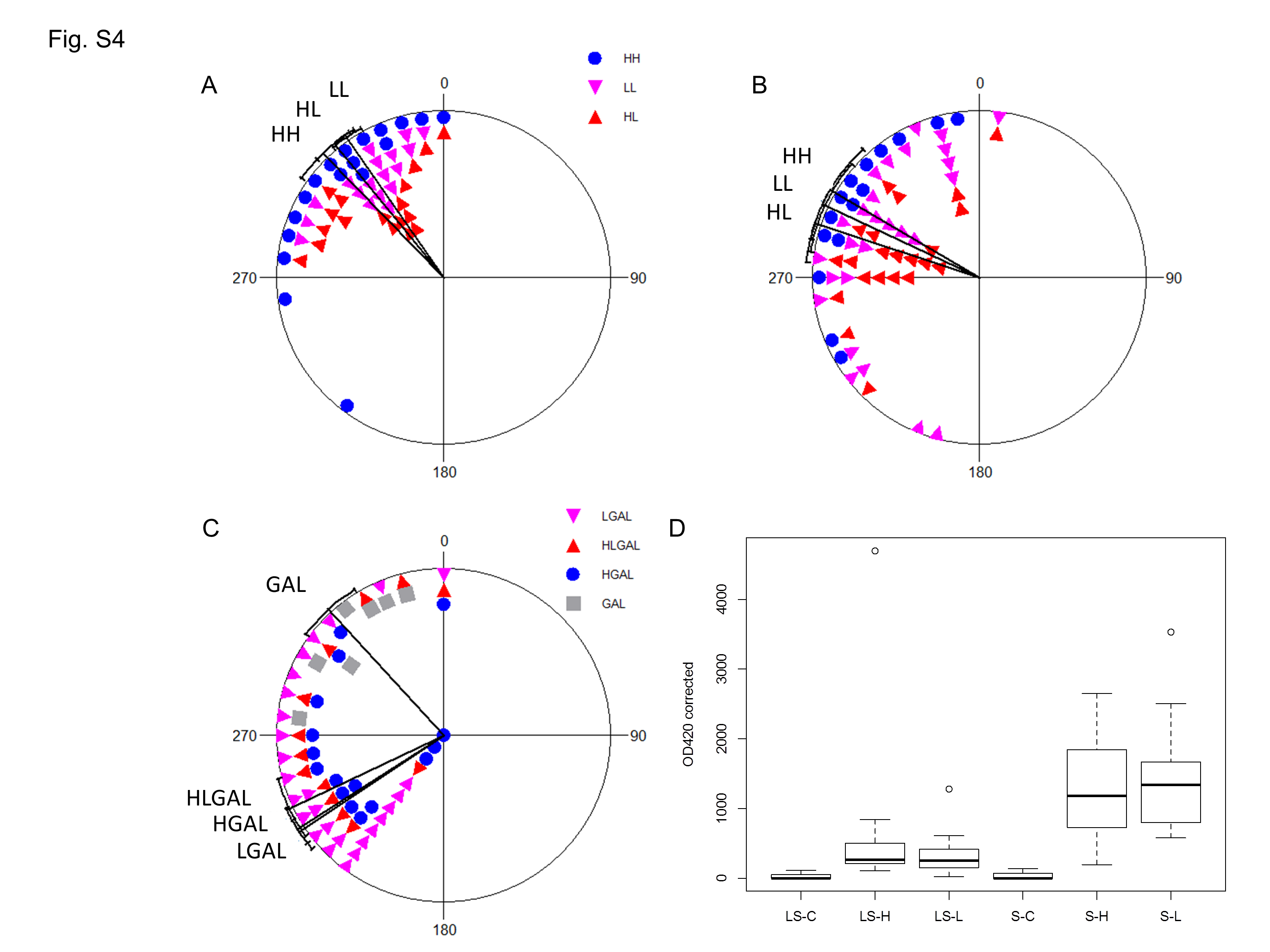

Supplement: Figure S4 — Circadian photosensitivity of cry variants. Behavioural phase delay to a 20 min light pulse administered at ZT15 of RENDE (A), CS (B) or transgenic (C) lines expressing CRYH, CRYL or both. Delays in hours (assigned negative values by convention) were converted to degrees (1 hr = 15°). No statistical difference was found in the delay response of flies expressing CRYH or CRYL (Watson-Williams F-test, RENDE F2,80 = 1.148 p = 0.322, CS F2,61 = 0.509 p = 0.604, Transgenic F2,209 = 1.111 p = 0.331). The lines represent the mean vectors (±95% confidence intervals). D. Yeast two hybrid testing of TIM-CRY interactions. OD420 normalized over OD720 of 20 yeast colonies expressing either L-TIM or S-TIM with CRYH or CRYL. No difference was found between the interaction of CRYH and CRYL with either L-TIM or S-TIM (L-TIM CRYH vs CRYL F1,38 = 1.038 p = 0.3146; S-TIM CRYH vs CRYL F1,38 = 0.06044 p = 0.8071). (TIF) [file pone.0086483.s004.tif]

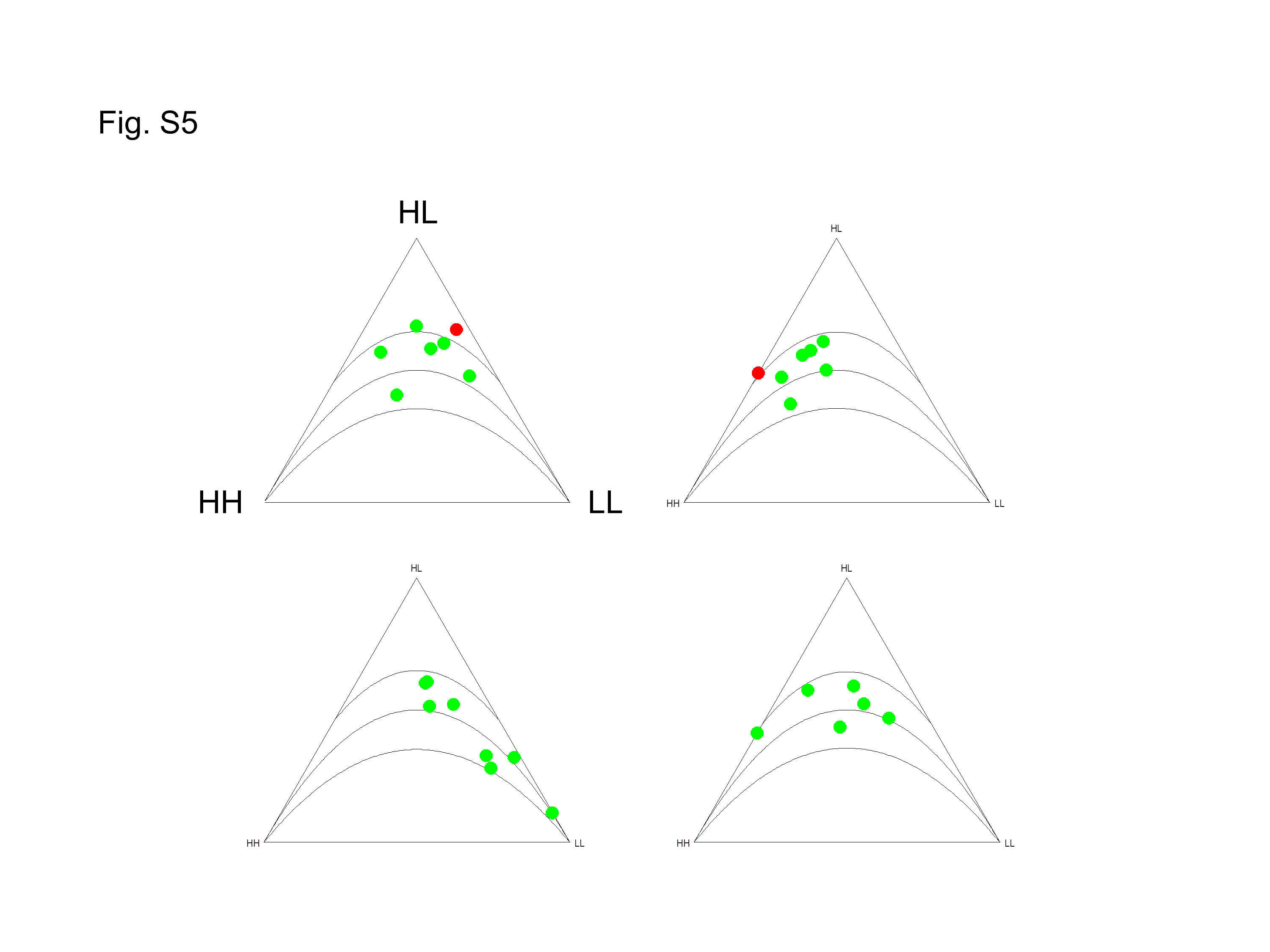

Supplement: Figure S5 — Change in genotype frequencies of the L232H SNP in population cages. Ternary plots showing the genotype frequency in each of the four population cages (same arrangement as in Fig. 5). Populations were genotyped 6 times during the experiment (16 months). Genotype frequencies under Hardy-Weinburg Equilibrium (HWE) are within the 95% limits and shown in green. Significant departures from HWE are shown in red. (TIF) [file pone.0086483.s005.tif]
