# Supplementary material for: Molecular Evolution of a Pervasive Natural Amino-Acid Substitution in Drosophila cryptochrome
Source: PLoS One. 2014 Jan 24;9(1):e86483. doi: 10.1371/journal.pone.0086483 (PMC3901690; doi:10.1371/journal.pone.0086483)
Supplement: Table S1 — Fly population samples. Geographical information, number of iso-female lines (N), and allele frequency. (DOC) [file pone.0086483.s006.doc]

## Table S1. Fly population samples.

|  | | | | | | | | | | |
| --- | --- | --- | --- | --- | --- | --- | --- | --- | --- | --- |
| Populations | | Location | Country of collection | Latitude | Longitude | Altitude (m) | Collection Date | N flies | CTT(L) frequency | CTA(H) frequency |
|  | SS | Haifa | Israel | 32.48N | 34.59E | 180 | 2002 | 42 | 0.5952 | 0.4048 |
|  | KNO | Knossos | Greece | 35.17N | 25.9E | 253 | 2002 | 44 | 0.6364 | 0.3636 |
|  | SAL | Salice | Italy | 40.23N | 17.58E | 34 | 2004 | 44 | 0.6591 | 0.3409 |
|  | RUT[[1]](#footnote-2) | Rutigliano | Italy | 40.56N | 16.54E | 100 | 2004 | 39 | 0.6026 | 0.3974 |
|  | FUL^a^,[[2]](#footnote-3) | Fulda | Germany | 50.33N | 9.40E | _ | 2002 | 7 | 0.29 | 0.71 |
|  | SLUG^a,b^ | Sluderno | Italy | 46.40N | 10.35 | _ | 2002 | 10 | NA | NA |
|  | CORST ^a,b^ | Bolzano | Italy | 46.30N | 11.21 |  | 2002 | 10 | NA | NA |
|  | BIT | Bitetto | Italy | 41.2N | 16.45E | 92 | 2004 | 43 | 0.5814 | 0.4186 |
|  | CAV^a^ | Cavarzere | Italy | 45.8N | 12.4E | 4 | 2004 | 38 | 0.4868 | 0.5132 |
|  | VIL | Villorba | Italy | 45.44N | 12.14E | 26 | 2004 | 9 | 0.5000 | 0.5000 |
|  | BOL | Bolzano | Italy | 46.30N | 11.21E | 262 | 2004 | 36 | 0.5139 | 0.4861 |
|  | COR-FRI | Corbières | Suisse | 46.39N | 7.6E | 728 | 2004 | 17 | 0.5588 | 0.4412 |
|  | BUR | Burgundy | France | 47.17N | 5.02E | 245 | 2000 | 17 | 0.5882 | 0.4118 |
|  | VNN | Wien | Austria | 48.12N | 16.22E | 151 | 2004 | 12 | 0.4583 | 0.5417 |
|  | HU | Houten | Netherlands | 52.1N | 5.10E | 2 | 2002 | 85 | 0.6588 | 0.3412 |
|  | LE | Leiden | Netherlands | 52.9N | 4.29E | -1 | 2000 | 16 | 0.6563 | 0.3438 |
|  | STO | Stockholm | Sweden | 59.19N | 18.4E | 44 | 2008 | 17 | 0.5882 | 0.4118 |
|  | KOR | Korpilahti | Finland | 62.1N | 25.33E | 120 | 2008 | 67 | 0.4925 | 0.5075 |
|  | HOJ | Højbjerg | Denmark | 56.7N | 10.12E | 2 | 2009 | 76 | 0.6513 | 0.3487 |
|  | MAR | Market  Harborough | UK | 52.28N | 0.55W | 93 | 2008 | 121 | 0.4793 | 0.5207 |
|  | KIL | Kibworth | UK | 52.32N | 0.59W | 110 | 2008 | 161 | 0.5776 | 0.4224 |
|  | GOT | Gothenburg | Sweden | 57.42N | 11.58E | 4 | 2008 | 35 | 0.4429 | 0.5571 |
|  | SP22 | Nijar | Spain | 36.58N | 2.12W | 356 | 2008 | 47 | 0.4787 | 0.5213 |
|  | SP28 | Jumilla | Spain | 38.28N | 1.19W | 510 | 2008 | 37 | 0.5676 | 0.4324 |
|  | SP38 | Requena | Spain | 39.29N | 1.6W | 692 | 2008 | 84 | 0.6012 | 0.3988 |
|  | SP52 | San Sadurni  D'anoia | Spain | 41.25N | 1.47E | 162 | 2008 | 65 | 0.5385 | 0.4615 |
|  | SP51 | La Rapita | Spain | 40.37N | 0.35E | 10 | 2008 | 71 | 0.6479 | 0.3521 |
|  | SP25+26 | Abaran | Spain | 38.12N | 1.24W | 400 | 2008 | 26 | 0.5769 | 0.4231 |
|  | SP35 | Fontanares | Spain | 37.46N | 3.43W | 634 | 2008 | 58 | 0.5517 | 0.4483 |
|  | SP14+15 | Algarrobo | Spain | 36.46N | 4.2W | 86 | 2008 | 28 | 0.7321 | 0.2679 |

1. An isofemale line from this population was analysed in the initial 10 allele survey shown in Fig.2. [↑](#footnote-ref-2)
2. This population was not included in the population analysis due to small number of isofemale lines. [↑](#footnote-ref-3)
